# Application of the antibiotic batumin for accurate and rapid identification of staphylococcal small colony variants

**DOI:** 10.1186/1756-0500-5-374

**Published:** 2012-07-24

**Authors:** Larisa N Churkina, Svetlana I Bidnenko, Guido Lopes dos Santos Santiago, Mario Vaneechoutte, Lilja V Avdeeva, Olga B Lutko, Nadya M Oserjanskaja

**Affiliations:** 1 Institute of Microbiology and Virology, National Academy of Sciences of Ukraine, Kyiv, Ukraine; 2 Institute of Traumatology and Orthopedics, Medical Academy of Sciences of Ukraine, Kyiv, Ukraine; 3 Laboratory Bacteriology Research, Department of Clinical Chemistry, Microbiology & Immunology, Faculty of Medicine & Health Sciences, Ghent University, Ghent, Belgium

**Keywords:** *Staphylococcus aureus*, MRSA, Batumin, Staphylococcal small-colony variants

## Abstract

**Background:**

*Staphylococcus aureus* is a major human pathogen causing significant morbidity and mortality. The *S. aureus* colonies in osteomyelitis, in patients with cystic fibrosis and patients with endoprosthesis rejection frequently have an atypical morphology, i.e. staphylococcal small-colony variants, which form a naturally occurring subpopulation of clinically important staphylococci. Identification of these small colony variants is difficult, because of the loss of typical phenotypic characteristics of these variants.

We wanted to improve and simplify the diagnosis of staphylococcal infection using a diagnostic preparation, consisting of 5 μg batumin paper disks. Batumin possesses a unique selective activity against all studied *Staphylococcus* spp., whereas all other species tested thus far are batumin resistant. We assessed the efficacy of the batumin diagnostic preparation to identify staphylococcal small colony variants, isolated from osteomyelitis patients.

**Findings:**

With the batumin diagnostic preparation, all 30 tested staphylococcal small-colony variants had a growth inhibition zone around the disk of minimum 25 mm, accordant with the inhibition zones of the parent strains, isolated from the same patients.

**Conclusions:**

The batumin diagnostic preparation correctly identified the small-colony variants of *S. aureus*, *S. haemolyticus* and *S. epidermidis* as belonging to the genus *Staphylococcus*, which differ profoundly from parental strains and are difficult to identify with standard methods. Identification of staphylococcal small-colony variants with the batumin diagnostic preparation is technically simple and can facilitate practical laboratory work.

## Findings

### Background

*Staphylococcus aureus* is a major human pathogen causing significant morbidity and mortality in both community and hospital acquired infections [1]. The prevalence of methicillin-resistant *S. aureus* is increasing, and treatment has become more difficult due to the emergence of multidrug – resistant strains [2-4]. Infections caused by *S. aureus* are predominantly acute [5-7]. However, *S. aureus* can also cause chronic disease with recurrent infections as in osteomyelitis [8], in airway infections of cystic fibrosis patients [9], or by colonizing tissues in endoprosthesis replacement, causing endoprosthesis rejection [10]. Agents of these infections present as atypical *S. aureus* forms, i.e. staphylococcal small-colony variants (SSCVs), which form a naturally occurring subpopulation of clinically important staphylococci and which can be isolated in pure culture.

Correct identification of bacterial small colony variants (SCVs), specifically those of the genus *Staphylococcus* (SSCVs), faces some difficulties caused by the loss of typical phenotypic characteristics of these variants. Moreover, these SSCVs grow slowly, and as a result form small colonies, with reduction in toxin production and delayed coagulase activity [11]. It may be possible to improve accuracy of identification and simplify the diagnostics using antimicrobial agents with selective action against staphylococci. To this purpose, the antibiotic batumin with unique activity against all studied species of the genus *Staphylococcus*[4,12], has been used for the development of a in house diagnostic preparation, which consists of disks impregnated with 5 μg of batumin. Previous results indicated the usefulness of the preparation for quick and reliable identification of isolates of the genus *Staphylococcus* with normal colony morphology [12-14]. As a result of successful testing of the batumin disks, a permit for the release of the new batumin diagnostic preparation was obtained and its application in clinical institutions of Ukraine was approved by The State Expert Center of the Ministry of Health of Ukraine (Protocol N P.09.01/03621 of September 6, 2001).

Here, we assessed the usefulness of the batumin diagnostic preparation for genus level identification of SSCVs.

### Results and discussion

Culture of the samples and identification of the isolates with normal colony morphology, indicated the presence of *Staphylococcus aureus*, *S. haemolyticus* and *S. epidermidis* (Table 1).

**Table 1:** Biochemical characteristics of staphylococcal small colony variants (SSCVs)

**Characteristic**	***S. aureus*18**	***S. aureus*71**	***S. aureus*127**	***S. aureus*143**	***S. aureus*187/1**	***S. aureus*187/2**	***S. aureus*218**	***S. aureus*280**	***S. aureus*297**	***S. aureus*389**	***S. aureus*505**	***S. aureus*531**	***S. aureus*544**	***S. aureus*601**	***S. aureus*1206**	***S. epidermidis*151**	***S. epidermidis*177**	***S. epidermidis*222**	***S. epidermidis*233/1**	***S. epidermidis*241**	***S. epidermidis*269**	***S. epidermidis*349**	***S. epidermidis*384**	***S. epidermidis*385**	***S. epidermidis*604**	***S. haemolyticus*201**	***S. haemolyticus*222/2**	***S. haemolyticus*242**	***S. haemolyticus*321**	***S. haemolyticus*606**
Sucrose^a^	-	-	-	-	-	-	-	-	+	+	-	+	+	-	+	-	+	+	-	-	+	-	-	+	+	+	+	-	+	+
Maltose^a^	-	-	-	±	-	+	-	±	+	+	-	+	+	-	+	+	+	-	-	±	-	-	-	+	-	-	-	-	+	-
D-mannitol^a^	-	-	-	-	-	-	+	-	+	+	+	-	+	-	+	-	-	-	-	-	+	-	-	-	-	+	-	-	+	+
D-mannose^a^	-	-	+	+	+	+	-	-	+	-	-	-	-	-	-	-	-	-	-	-	-	+	+	-	-	+	+	-	-	+
D-trehalose^a^	+	+	-	+	+	+	+	+	-	-	-	+	+	+	+	-	-	-	+	-	-	-	+	-	+	+	+	+	-	-
Lactose^a^	-	-	+	-	+	+	+	-	-	-	-	-	-	+	-	+	+	+	-	-	-	-	-	+	-	-	-	-	+	+
D-fructose^a^	+	+	-	+	+	-	-	-	+	+	+	+	+	+	-	+	+	+	-	-	-	+	-	-	-	+	+	-	-	-
D-glucose^a^	+	+	+	+	+	+	+	+	+	+	+	+	+	+	+	-	-	-	+	+	+	-	-	-	-	+	+	+	-	+
Nitrate reductase	-	-	+	-	-	+	-	+	+	-	-	-	-	-	-	-	-	-	+	+	-	+	+	+	-	-	-	-	-	+
Phospatase	-	-	-	-	-	+	+	-	-	+	-	+	-	-	+	-	-	+	+	-	-	-	-	+	+	-	-	-	+	+
Urease	-	-	-	-	-	-	+	-	-	-	-	+	-	+	+	-	+	+	+	+	-	-	-	+	-	-	-	+	-	-
Coagulase (rabbit plasma)	-	-	±	-	-	-	±	-	-	±	-	-	-	±	-	-	-	-	-	-	-	-	-	-	-	-	-	-	-	-
Lecithinase	-	-	-	±	±	-	-	-	-	-	-	-	-	-	±	-	-	-	-	-	-	-	-	-	-	-	-	-	-	-
Hemolysis	-	-	-	-	-	-	+	-	-	+	-	+	-	-	-	±	±	-	-	-	-	-	-	-	-	+	+	±	+	+

All the colonies of the SSCVs were colorless, oxidase and catalase negative.

Gram staining revealed the presence of Gram-positive polymorphic cocci, both for the parental strains and the SSCVs.

For SSCVs, lecithinase activities were absent with exception of the *S. aureus* strains 143, 187/1, 1206 (weakly lecithinase positive) and coagulase activities were absent with exception of the *S. aureus* strains 127, 218, 389, 601 (weakly coagulase positive) (Table 1).

Although the tube coagulase test is considered as the gold standard in *S. aureus* identification [15], this study indicates that it is unreliable in the identification of SSCVs. The altered metabolism of SSCVs for carbohydrates, such as xylose, raffinose, melibiose, sucrose, maltose and lactose, as established here (Table 1), confirms previous findings [11].

Moreover, isolation and identification of atypical forms is further complicated by the fact that they are auxotrophic on haemin, amino acids and fatty acids [16]. To produce normal growth, various supplements, such as haemin and menadione, have to be added to the culture media.

The 17 SSCVs studied for auxotrophy could be classified into haemin dependent (*S. aureus* 18, 127, 187/2, 218, 280, 505, and *S. epidermidis* 233/1, 269, 349, 604), menadione dependent (*S. aureus* 187/1, 389 and *S. epidermidis* 151, 384, 385, 177) and thymidine dependent (only *S. aureus* 71).

Because the SSCVs lack a number of characteristics which otherwise enable identification with established phenotypic tests, we evaluated whether batumin impregnated disks were able to correctly identify the 30 SSCV isolates. The presence of a growth inhibition zone of 17 mm or more around the disk was considered as evidence that the isolate belonged to the genus *Staphylococcus*, because we previously showed that the inhibition zones for 658 *S. aureus*, 152 *S. epidermidis*, 42 *S. saprophyticus* isolates and all isolates from 7 other *Staphylococcus* spp. were 17–29 mm, whereas all other Gram-positives (*Dermacoccus* spp. (5 isolates), *Enterococcus faecalis* (84), *Kociura* spp. (17), *Kytococcus* spp. (3), *Microcococcus* spp. *(84)Planocococcus* spp. (1), *Streptococcus pyogenes* (36) and viridans streptococci (30)) gave no inhibition zones, except for 10 isolates of *Corynebacterium xerosis*, which gave zones of 10 mm [12].

**Figure 1:**
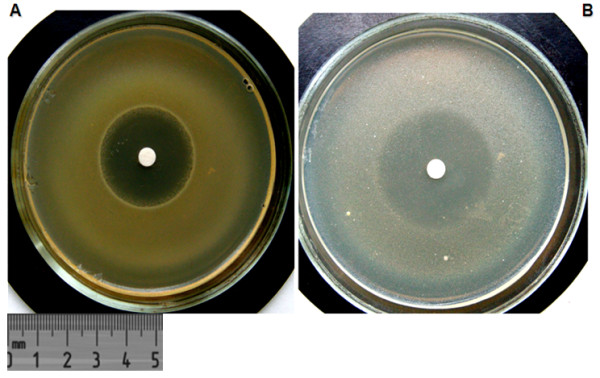
Identification with batumin of a) staphylococci with normal colony morphology and b) staphylococcal small colony variants.

All 30 SSCVs gave a growth inhibition zone around the diagnostic disk of 25 mm and more, which can be interpreted as evidence that the isolates belong to the genus *Staphylococcus* (Figure 1).

Reliability of identification of the SSCVs by batumin impregnated disks as staphylococci was confirmed by molecular identification with tRNA-intergenic spacer length polymorphism analysis, shown previously to enable identification of staphylococci [17-19].

### Conclusions

In conclusion, batumin impregnated disks correctly identified the SCVs of *S. aureus, S. haemolyticus and S. epidermidis* as staphylococci, although the SCVs differ profoundly from parental strains and are difficult to identify with standard methods. Identification of SSCVs by batumin is technically simple and can facilitate practical laboratory work.

### Methods

Bone and muscle tissue biopsies, from patients with osteomyelitis in the Kyiv Institute of Traumatology and Orthopedics, Medical Academy of Sciences of Ukraine, were received as a part of standard medical care and this was approved by Ethics Committee of the Institute of Traumatology and Orthopedics, Medical Academy of Sciences of Ukraine (Protocol N2 of December 6, 2011).

The bone and muscle tissue biopsies were cultured onto Columbia agar with 5% washed sheep erythrocytes. A total of 30 staphylococcal SSCVs, i.e. slowly growing pinpoint colonies after 24–48 hours of incubation, were obtained as subpopulations of staphylococci with normal colony morphology. Identification of the staphylococci was carried out according to standard methods [20].

SSCVs were isolated on Columbia agar with 5% sheep blood, as pinpoint colonies (0.1-0.3 mm) after 48 hours of aerobic incubation at 37 °C, among the more numerous colonies (2–3 mm) with normal *Staphylococcus* morphology, considered as the staphylococcal parental isolates.

After re-isolation, colony morphology and pigmentation were observed on pepton agar plates (10 g pepton agar, 5 g yeast extract, 5 g NaCl, 1 g glucose and 15 g agar, per L of distilled water [21]. Acid production from carbohydrates and enzymatic activities were studied with API-Staph galleries (Biomérieux, Boxtel, The Netherlands). Fermentation of glucose was determined according to Hugh and Leifson [22], catalase activity by mixing a colony with a drop of 3% H_2_O_2_, oxidase activity according to Faller and Schleifer [23], coagulase activity according to Sperber and Tatini [24], lecithinase activity according to the method of Akatov and Zueva [25], and hemolytic activity on agar supplemented with 5% (vol/vol) washed rabbit erythrocytes.

For auxotrophy determination, the isolates were plated on Müller-Hinton agar (MHA) with a 2 μg haemin disk, a 10 μg menadione disk [26] or a 1 μg thymidine disk.

Batumin is commercially available from Santa Cruz Biotechnology (Santa Cruz, CA) or Enzo Life Sciences (Antwerp, Belgium).

## Abbreviations

SSCV, Staphylococcal small-colony variants; SCV, Small-colony variants; MHA, Müller-Hinton agar.

## Competing interests

The authors declare that no competing interests exist.

## Authors' contributions

SB, OL, NO: isolation of staphylococcal small colony variants (SSCVs) from osteomyelitis patients and their identificaton. LA: study of Sensitivity of staphylococcal clinical strains to wide spectrum of antibiotics. MV, GLdSS: part of the testing, providing with strains, identification of strains, writing. LC - Identification of small colony variants by batumin, article writing. All authors read and approved the final manuscript.
